# Experiences of men with breast cancer: an exploratory focus group study

**DOI:** 10.1038/sj.bjc.6601305

**Published:** 2003-11-11

**Authors:** B G Williams, R Iredale, K Brain, E France, P Barrett-Lee, J Gray

**Affiliations:** 1 Institute of Medical Genetics, University of Wales College of Medicine, Heath Park, Cardiff CF14 4XN, UK; 2 Institute of Medical Genetics, Cardiff and Vale NHS Trust, Heath Park, Cardiff CF14 4XN, UK; 3 Velindre Hospital, Cardiff and Vale NHS Trust, Cardiff, UK

**Keywords:** male breast cancer, patient support, gender, focus group

## Abstract

Management and care of men with breast cancer is based on that developed for women. Our study reports that men have specific issues regarding certain aspects of their breast cancer experience, including diagnosis, disclosure, support and gender-specific information, and offers suggestions for improved patient care.

Breast cancer in men is a rare condition, accounting for only 1% of all cases of breast cancer (Sasco 1993; Young *et al*, 2000). In the UK, around 300 men are diagnosed with the condition every year (Cancer Research UK, 2002). While the relatively common nature of female breast cancer has resulted in a high level of general awareness about the condition, male breast cancer is still a comparatively unknown entity, both by the general public and by healthcare and social care professionals. There is very little research on the management and care of male breast cancer, and that which exists is often extrapolated from research about breast cancer in women (Fenlon, 1996; reviewed in Ravandi-Kashani and Hayes, 1998; Bunkley *et al*, 2000).

An earlier pilot study conducted by the authors suggested that men with breast cancer have issues and concerns specific to them, which include delay in diagnosis, shock, stigma, altered body image, lack of emotional support, provision of inappropriate information and perception of causal factors (France *et al*, 2000). The results presented in this paper derive from the first part of a multistage project that uses a variety of methods to investigate patient needs and current management practice with a larger group of men across the UK. The first stage of the project employs focus group methodology to explore the experiences of men who have had a breast cancer diagnosis and to compare them with those of women with breast cancer and the opinions of healthcare professionals who care for both patient groups. In this short communication, we identify some of the important issues for men coping with breast cancer.

## METHODS

Stage one of this project involved a series of four focus group discussions in South Wales. The groups comprised of two groups of men with breast cancer identified from a regional oncology unit: one group of women with breast cancer attending local support groups and one group of healthcare professionals, such as breast surgeons, breast care nurses and oncologists, currently treating breast cancer patients. A total of 27 people, all from South Wales, participated in the focus group discussions. Ethical approval for the study was obtained from a multi-centre research ethics committee.

The basis of each discussion was a semi-structured topic guide developed from the literature, earlier research and discussions with colleagues (Barbour and Kitzinger, 1999). The main topics under discussion for each group are outlined in Table 1

**Table 1:** Main discussion topics within each focus group

**Men**	**Women**	**Healthcare professionals**
Patient experiences	Patient experiences	Perceived differences between men and women
Delays in diagnosis	Support (formal and informal)	Interaction with patients
Information received	Information received	Information provided
Support (formal and informal)	Perceptions of impact of condition on men	Gender-specific resources

.

All focus groups were tape recorded and transcribed verbatim. The transcripts were analysed by hand using thematic analysis and the main themes discussed among the authors for validation. Four themes were identified – diagnosis, disclosure, support and gender-specific information.

## RESULTS

### Diagnosis

Men with breast cancer often delay visiting their GP between 6 and 9 months (Goss *et al*, 1999). However, participants in our study did not describe such a delay. Interestingly, this may be due to the role of their wives or partners in prompting the referral. One man in particular was finally referred when his wife insisted on a second opinion after his GP failed to recognise his symptoms: ‘She [wife] said – “You've got to go. I've made an appointment”‘ (Male).

When receiving a breast cancer diagnosis, healthcare professionals described men as reacting stoically, concerned with practical issues. Women were perceived as reacting more emotionally. These reactions in turn, influenced how the healthcare professional dealt with the patient:‘I'm not as sensitive and sort of tender loving care in my voice [with men] as I am with women … I'm much more matter of fact and don't make a big issue out of it’ (Breast care nurse).

### Disclosure

All the men stated that they were open about their diagnosis of breast cancer: ‘I don't discuss it openly with anybody unless it is directed at me’ (Male).

However, some men were concerned with their appearance. For example, one man had stopped swimming, while another refused to remove his shirt at the beach. One man was aware that people would stare at his scar, but ignored them: ‘I’ve been abroad and sunbathed. People do look, they do look. People don't care. Only you care. Nobody else cares. After a while you get to know that. They just look at you and say ‘Oh’ (Male).

### Support

For most men, their main source of support throughout their experience was their partner. The only formal support received was provided by the breast care nurse, whose efforts and advice was valued.

Healthcare professionals reported that they often set up support links for women and both they and women in the focus groups said they felt it was worthwhile. However, there was a perception by some healthcare professionals that a similar network for men would not work. Only one man was given the opportunity to contact another man with breast cancer, but all the men felt it could be useful for those who were undergoing treatment to have the opportunity to talk to another man: ‘One of the worst things was the fact there weren't any men I could go to’ (Male).

Generally, men had appreciated the opportunity to attend the focus group, which had allowed them to talk and share experiences with other men. Women gave mixed views as to whether they would be happy for men to attend a support group: ‘I don't think women would naturally turn to a man and discuss it like you would to the next woman sitting alongside you’ (Female).

Men themselves would not want to attend mixed groups because they thought they would be less likely to disclose in the presence of women who, in their opinion, would have been more affected by their diagnosis.

### Gender-specific information

Many men were disappointed at the lack of information on breast cancer that was relevant to men. The literature they received covered topics that were specific to women. For example, most leaflets discussed issues such as menstruation, breast reconstruction and bra fittings.

Interestingly, the men in our study did not want separate leaflets and booklets devoted to male breast cancer, but rather separate sections on issues relevant to men incorporated into existing material: ‘If there was just one little paragraph for male breast cancer…’ (Male).

Healthcare professionals also agreed that supplementary information available in existing leaflets and booklets would be useful to male patients, such as side effects of adjuvant hormonal therapy including loss of libido. Before the mastectomy operation, patients are sometimes shown pictures of a female mastectomy. Men, women or healthcare professionals considered this inappropriate for men and healthcare professionals discussed the possibility of providing a photograph of a male mastectomy. Some men commented that this would be more reassuring – ‘I think a male photograph … that way you are showing someone this is what is going to be you after the operation’ (Male).

## DISCUSSION

Our study highlights some important issues in the management and care of men with breast cancer and offers suggestions for improvement. Firstly, men reported visiting the GP promptly on discovering their symptom because of their partner's insistence. This highlights the important role a (female) partner plays both in prompt diagnosis (Seymour-Smith *et al*, 2002) and throughout men's breast cancer experience, which should be encouraged and supported.

Although men perceived the breast care nurse as an important source of support, most men would appreciate the opportunity to talk to another man with breast cancer on a one-to-one basis. Breast care nurses currently provide support links for women, but did not think men would be interested in a similar scheme. These findings suggest that healthcare professionals tend to adopt a more pragmatic approach when dealing with male breast cancer patients by focusing on their practical rather than emotional needs. Some consideration should be given to matching schemes for men.

Finally, this study confirms that there is a paucity of relevant information available to men (Peate, 2001), many of whom may have specific concerns, for example personal appearance following breast cancer diagnosis and treatment. Rather than developing completely new information sources for men, a supplementary section within the existing leaflets and booklets on areas specific to men was considered to be useful. Incorporating a photograph of a male mastectomy into existing patient materials would be a simple and cost-effective way of improving care for men.

This study has highlighted some important considerations for men with breast cancer and offers some suggestions for change. Limitations of the study include the small numbers of participants and possible self-selection to participate in focus group research. The next stage will be to collect survey data from a larger and representative sample of men across the UK about their psychological and informational needs in relation to their breast cancer.

## References

[bib1] Barbour RS, Kitzinger J (1999) Developing Focus Group Research. London: Sage

[bib2] Bunkley DT, Robinson JD, Bennett Jr NE, Gordon S (2000) Breast Cancer in men: emasculation by association? J Clin Psychol Med Settings 7: 91–97

[bib3] Cancer Research UK (2002) Cancer Stats: Incidence – UK. London: Cancer Research UK

[bib4] Fenlon D (1996) Endocrine therapies for breast cancer. In Breast Cancer Nursing, Denton S (ed) pp 104–121. London: Chapman & Hall

[bib5] France L, Michie S, Barrett-Lee P, Brain K, Harper P, Gray J (2000) Male cancer: a qualitative study of male breast cancer. Breast J 9: 343–348 10.1054/brst.2000.0173 14965759

[bib6] Goss PE, Reid C, Pintilie M, Lim R, Miller N (1999) Male breast carcinoma. A review of 229 patients who presented to the Princess Margaret Hospital during 40 years: 1955 – 1996. Cancer 85: 629–639 10091736 10.1002/(sici)1097-0142(19990201)85:3<629::aid-cncr13>3.0.co;2-v

[bib7] Peate I (2001) Caring for men with breast cancer: causes, symptoms and treatment. Br J Nursing 10: 975–981 10.12968/bjon.2001.10.15.5262 11923732

[bib8] Ravandi-Kashani F, Hayes TG (1998) Male breast cancer: a review of the literature. Eur J Cancer 34: 1341–1347 9849414 10.1016/s0959-8049(98)00028-8

[bib9] Sasco AJ (1993) Epidemiology of male breast cancer. Int J Cancer 53: 538–549 8436428 10.1002/ijc.2910530403

[bib10] Seymour-Smith S, Wetherell M, Phoenix A (2002) ‘My Wife Ordered Me to Come’!: a discursive analysis of doctors' and nurses' accounts of men's use of general practitioners. J Health Psychol 7: 253–267 22114249 10.1177/1359105302007003220

[bib12] Young IE, Kurian KM, Mackenzie MAF, Kunkler IH, Cohen BB, Hooper ML, Wyllie AH, Steel CM (2000) The CAG repeat within the androgen receptor gene in male breast cancer patients. J Med Genet 37: 139–140 10712104 10.1136/jmg.37.2.139 PMC1734511

